# 超亲水印迹石墨烯气凝胶制备及其精准识别尿液中肿瘤标志物

**DOI:** 10.3724/SP.J.1123.2025.05021

**Published:** 2026-02-08

**Authors:** Mingwei WANG, Tao TIAN, Ligai BAI, Dandan HAN, Hongyuan YAN

**Affiliations:** 1. 药物化学与分子诊断教育部重点实验室，河北大学化学与材料科学学院，河北 保定 071002; 1. Key Laboratory of Medicinal Chemistry and Molecular Diagnosis of Ministry of Education，College of Chemistry and Materials Science，Hebei University，Baoding 071002，China; 2. 河北省公共卫生安全重点实验室，河北大学公共卫生学院，河北 保定 071002; 2. Hebei Key Laboratory of Public Health Safety，College of Public Health，Hebei University，Baoding 071002，China; 3. 河北省分析科学技术重点实验室，河北大学药学院，河北 保定 071002; 3. Hebei Key Laboratory of Analytical Science and Technology，College of Pharmaceutical Science，Hebei University，Baoding 071002，China

**Keywords:** 亲水印迹树脂, 石墨烯气凝胶, 水相识别, 固相萃取, 肿瘤标志物, 高效液相色谱, hydrophilic imprinted resin, graphene aerogel, specific recognition, solid-phase extraction, tumor biomarkers, high performance liquid chromatography （HPLC）

## Abstract

近年来，分子印迹聚合物在分析检测和疾病早期诊断领域展现出显著潜力，但其实际应用仍面临传统制备方法的诸多限制，包括有机溶剂依赖性强、水相识别能力弱以及吸附容量低等问题。针对这些挑战，本研究将亲水性树脂与石墨烯气凝胶复合，在水相体系下成功制备了超亲水印迹石墨烯气凝胶材料（HMIR-GA），显著提升了分子印迹材料的水相识别能力和吸附能力。本研究系统优化了制备参数，最终得到了水相识别能力强和吸附容量高的HMIR-GA。吸附试验结果表明，HMIR-GA对肿瘤标志物5-羟吲哚乙酸表现出优异的吸附性能和显著的特异识别能力（印迹因子可达8.8），能够有效区分待测组分与共存干扰物质。此外，制备的HMIR-GA对5-羟吲哚乙酸的回收率显著优于商业化吸附剂。将制备的新型HMIR-GA作为管尖固相萃取吸附剂，对关键萃取参数进行优化，在最优萃取条件下结合液相色谱开发了一种高效、灵敏的生物样品中痕量肿瘤标志物检测的新方法，检出限低至3.7 ng/mL，日内和日间精密度分别为2.9%和4.1%（*n*=6），加标回收率为75.7%~92.5%，且相对标准偏差（RSD）≤3.4%，可以实现神经内分泌肿瘤的早期诊断。本研究为功能化亲水分子印迹材料的设计与开发提供了新的研究思路和技术参考，也为生物样品中肿瘤标志物的精准分离和高灵敏检测提供了可靠技术方案。

## Abstract (en)

In recent years， molecularly imprinted polymers have shown considerable promise in analytical detection and early diagnosis of diseases due to their high selectivity and specificity. Nevertheless， the practical implementation of these methods is still restricted by several intrinsic limitations associated with traditional synthesis approaches， including a strong reliance on organic solvents， poor recognition efficiency in aqueous media， and low adsorption capacity. To overcome these challenges， this study presents an innovative strategy that integrates superhydrophilic resin with graphene aerogel （GA）， resulting in successful fabrication of a superhydrophilic molecularly imprinted resin-GA composite （HMIR-GA） via surface in situ polymerization in water. The resulting HMIR-GA exhibited a significantly enhanced adsorption capacity and improved recognition performance in aqueous environments towards tumor biomarker. Characterization of the HMIR-GA was performed using Fourier transform infrared （FT-IR） spectroscopy， scanning electron microscopy （SEM）， nitrogen adsorption-desorption analysis， and contact angle measurements. FT-IR spectra revealed that the broad peak at 3 400 cm^-1^ can be ascribed to the formation of –OH associations. The absorption peak at 1 724 cm^-1^ corresponds to the stretching vibration peak of C=O on the surface of graphene oxide （GO）. The absorption peaks at 1 602 cm^-1^ and 1 462 cm^-1^ are assigned to the C=C stretching vibration peaks of resorcinol. During the reaction process， due to the reduction effect of ammonia water， the C=O on graphene oxide is reduced. The characteristic peak at 1 069 cm^-1^ is induced by the stretching vibration of C-O-C， representing the formation by the reaction between resorcinol and hexamethylenetetramine. These characteristic peaks clearly demonstrate that the HMIR have been successfully incorporated into the graphene aerogel. The FT-IR results confirm the successful synthesis of HMIR-GA. SEM reveals that the surface of graphene oxide exhibits a wrinkled lamellar structure. In contrast， the fabricated HMIR-GA and superhydrophilic molecularly non-imprinted resin-GA composite （HNIR-GA） display a loose and porous architecture， indicating that the synthesized HMIR has been successfully grown onto the graphene aerogel. The porous structure is conducive to the rapid adsorption of 5-hydroxyindoleacetic acid （5-HIAA）， which is beneficial for enhancing the performance of relevant applications. The Brunauer-Emmett-Teller （BET） specific surface areas of HMIR-GA and HNIR-GA are 95.1 m²/g and 44.5 m²/g， respectively. The pore volumes are 0.31 cm³/g and 0.20 cm³/g， respectively. In comparison with HNIR-GA， HMIR-GA possesses a larger specific surface area and pore volume， which is conducive to enhancing its adsorption capacity for 5-HIAA. To evaluate the hydrophilicity of HMIR-GA and HNIR-GA， contact angle measurements were performed. The results showed that when water droplets were placed on the surfaces of these two materials， they rapidly spread and fully wetted the surfaces within 0.07 s， indicating that HMIR-GA and HNIR-GA exhibited superior hydrophilic properties. This enhanced hydrophilicity facilitates the effective adsorption and extraction of the tumor biomarker 5-HIAA from urine samples. Static and competitive adsorption experiments revealed that HMIR-GA has a strong affinity for 5-HIAA. The evaluation of adsorption kinetics was carried out by employing both the pseudo-first-order and pseudo-second-order models， indicating a better fit with the pseudo-second-order model （*R*²=0.999 3）， suggesting that chemisorption is the dominant mechanism. Furthermore， equilibrium adsorption data were analyzed using the following models—Langmuir， Freundlich， Temkin， and Dubinin-Radushkevich （D-R）. The best fit was achieved with the Freundlich isotherm model （*R*²≥0.985 2）， indicating multilayer adsorption on heterogeneous surfaces. A highly sensitive method for precise determination of 5-HIAA was established by employing HMIR-GA as a pipette tip solid-phase extraction adsorbent coupled with high performance liquid chromatography. The calibration curve exhibited excellent linearity across the mass concentration range of 0.02–40.0 μg/mL （*r*=0.999 8）. The limits of detection （LOD） and quantification （LOQ） were 3.7 ng/mL and 12.3 ng/mL， respectively， based on signal-to-noise ratios of 3 and 10. Method accuracy was verified through recovery tests at spiked mass concentrations of 0.1， 1.0， and 10.0 μg/mL， yielding recoveries between 75.7% and 92.5% with relative standard deviations （RSDs） below 3.4%. Precision assessments via intra-day and inter-day tests yielded RSDs of 2.9% and 4.1%， respectively （*n*=6）. Finally， the developed method was applied for the determination of 5-HIAA levels in real urine samples. This work not only provides a robust and environmentally friendly strategy for the fabrication of functionalized molecularly imprinted polymers but also shows great promise for clinical applications， offering crucial technical support for early diagnosis of gastroenteropancreatic neuroendocrine tumors.

胃肠胰腺神经内分泌肿瘤（GEP-NETs）作为一类罕见肿瘤，起源于胃肠道及胰腺的神经内分泌细胞，其通常生长缓慢，但有时可能分泌过多激素，引发多种临床表现，如腹部不适、消化问题、体重下降和低血糖等^［1］^。GEP-NETs的罕见性和高度异质性使其症状不典型且复杂，给诊断和治疗带来挑战。通过对肿瘤标志物的精准检测，可以有效识别早期且微小的癌症病变，并为及时采取干预措施提供依据，从而显著提高患者生存率和预后效果^［2，3］^。因此，研究特征性肿瘤标志物对于GEP-NETs的筛查、诊断和防治具有重要意义。研究表明尿液中5-羟吲哚乙酸（5-HIAA）水平升高是GEP-NENs的显著生化指标，且在患者中表现显著，在临床诊断中具有重要参考价值^［4］^。因此，开发一种精准快速的5-HIAA检测方法，对于GEP-NENs的早期识别和诊断至关重要。

肿瘤标志物的精准检测是深入理解生理和病理过程的基础和关键，并在疾病早期诊断、治疗监测及预后评估中具有重要意义。目前，5-HIAA检测方法主要包括化学检测法、荧光检测法、免疫分析法、液相色谱-质谱联用法和高效液相色谱法（HPLC）等^［5-9］^。由于其高效的分离能力、快速的分析速度、简便的操作流程以及相对较低的成本，HPLC在肿瘤标志物的研究中占据重要地位，具有较好的临床应用潜力^［10，11］^。然而，由于生物样品中肿瘤标志物浓度较低且基质成分复杂，检测过程中易受干扰，检测难度较大。因此，亟须开发高效的样品前处理技术，对生物样品中肿瘤标志物进行净化富集以实现其准确检测。

分子印迹材料（molecularly imprinted polymers， MIPs）是基于“分子识别”原理选择性富集与模板分子结构相似的目标物，可以同时实现复杂样品中痕量待测组分的净化和富集^［12-16］^。然而，采用传统方法制备的分子印迹材料在非极性或弱极性体系中展现出良好的选择性，但在极性体系尤其是水相体系中识别效率明显下降^［17］^。此外，传统印迹材料的吸附容量有限，限制了其在复杂生物样品中的应用。

亲水酚醛树脂材料是一类具有丰富亲水官能团、空间三维网状结构和较高吸附容量等多重功能的高分子聚合物，其原料来源广泛、合成工艺简便，所得吸附剂具有优良的化学稳定性、机械强度及吸附性能^［18-20］^。同时，石墨烯气凝胶材料由于比表面积大、吸附位点丰富、疏松多孔以及良好的理化稳定性，近年来在样品前处理领域中受到广泛关注^［21，22］^。其疏松多孔结构能够有效促进目标分子快速进入并结合至活性位点，显著提升材料的识别效率和吸附能力。

本研究将分子印迹、亲水酚醛树脂与石墨烯气凝胶相结合，成功构建了亲水分子印迹树脂-石墨烯气凝胶复合材料（hydrophilic molecularly imprinted resin-graphene aerogel， HMIR-GA）。该材料兼具优异的亲水性与多吸附位点，可显著提升印迹材料在水相中的识别能力与吸附容量。同时，以HMIR-GA为吸附剂，同时结合HPLC技术，开发了一种用于生物样品中肿瘤标志物的精准快速检测新方法，成功应用于实际尿液样品中5-HIAA的有效净化与精准检测，为GEP-NENs的早期筛查和诊断提供了新思路与技术支撑。

## 1 实验部分

### 1.1 仪器、试剂与材料

Vanquish Core高效液相色谱仪和Sorvall^TM^ Stratos^TM^离心机（美国赛默飞科技有限公司）；Phenom Pro扫描电子显微镜（荷兰飞纳公司）；Vertex70傅里叶变换红外光谱仪（德国布鲁克公司）；TristarⅡ3020全自动比表面积和孔隙分析仪（美国Quantachrome仪器有限公司）；Millipore超纯水系统（德国默克科技公司）。

氧化石墨烯（Ⅱ型粉末）购自济宁利特纳米技术有限公司。间苯二酚（分析纯）购自上海麦克林科技有限公司。乌洛托品（分析纯）、*N*-（1-萘基）乙二胺二盐酸盐（分析纯）、5-羟吲哚乙酸（标准品，纯度≥98%）、甲砜霉素（标准品，纯度≥99%）、香草扁桃酸（标准品，纯度≥99%）、盐酸四环素（标准品，纯度≥99%）、氟苯尼考（标准品，纯度≥99%）、甲酸（色谱纯）均购自上海阿拉丁生化科技有限公司，聚乙烯醇（分析纯）购自天津市科密欧化学试剂有限公司。甲醇（色谱纯）购自上海星可生化有限公司。

### 1.2 HMIR-GA的制备

精确称取150 mg氧化石墨烯，加入15.0 mL水，超声分散均匀，随后加入3.0 mmol间苯二酚、1.5 mmol乌洛托品，40 ℃下加热搅拌2 h。加入0.09 mmol虚拟模板*N*-（1-萘基）乙二胺二盐酸盐，40 ℃下继续加热搅拌2 h后加入200 mg聚乙烯醇，升温至90 ℃继续搅拌1 h，随后转移至内衬高压反应釜中120 ℃反应2 h。产物冷却后先经冷冻干燥完全，采用甲醇-乙酸（9∶1，体积比）溶液对模板进行完全去除，然后用去离子水洗至中性。最终，通过冷冻干燥处理，成功制备得到HMIR-GA。

非印迹材料HNIR-GA的制备过程除不加入虚拟模板外，其余步骤与HMIR-GA制备过程相同。

### 1.3 HMIR-GA的吸附性能考察

为获得最佳印迹效果，系统优化了合成过程中间苯二酚与乌洛托品的物质的量之比、模板用量、印迹时间及聚乙烯醇用量，采用5-HIAA的吸附量作为性能评估指标。

HMIR-GA吸附量测定：称取3.0 mg HMIR-GA于10 mL聚丙烯离心管中，加入20 μg/mL 5-HIAA标准溶液2.0 mL，于25 ℃、300 r/min条件下吸附4 h。吸附结束后以4 500 r/min离心，取上清液经0.22 μm滤膜过滤后进行浓度测定，计算吸附量（*Q*ₑ， μg/mg），公式如下^［10，12］^：


*Q*_e_=（*C*_0_-*C*_e_）×*V*/*W* （1）

其中，*C*_0_和*C*_e_分别为吸附前后5-HIAA的质量浓度（μg/mL），*V*为溶液体积（mL），*W*为吸附剂HMIR-GA的质量（mg）。

吸附动力学实验：分别称取3.0 mg HMIR-GA与HNIR-GA于10 mL的聚丙烯离心管中，加入20 μg/mL 5-HIAA标准溶液2.0 mL，在25 ℃下振荡不同时间（1~720 min），吸附结束后同样离心并过滤，按公式（1）计算吸附量^［10，12］^。

吸附等温线实验：分别称取3.0 mg HMIR-GA与HNIR-GA于10 mL的聚丙烯离心管中，加入质量浓度为2~100 μg/mL的5-HIAA标准溶液2.0 mL，在不同温度（25、35、45 ℃）下振荡4 h，吸附后离心、过滤、测定，计算吸附量^［10，12］^。

竞争性吸附实验：称取3.0 mg HMIR-GA与HNIR-GA分别放至10 mL离心管中，加入质量浓度为20 μg/mL的混合标准溶液（含5-HIAA、甲砜霉素、香草扁桃酸、盐酸四环素、氟苯尼考）2.0 mL，25 ℃下振荡4 h，吸附结束后离心过滤并进样分析，按公式（1）计算吸附量^［10，12］^。通常采用印迹因子（IF）评估印迹材料的选择性识别能力，其计算公式如下：

IF=*Q*_HMIR-GA_/*Q*_HNIR-GA_ （2）

其中，*Q*_HMIR-GA_和*Q*_HNIR-GA_分别为HMIR-GA与HNIR-GA的吸附量。

### 1.4 HMIR-GA-管尖-固相萃取（HMIR-GA-PT-SPE）过程

将采集的6份尿液样品放入冰箱（-20 °C）经3次冻融循环后，以10 000 r/min离心5 min，去除沉淀后用0.22 μm滤膜过滤，然后取1.0 mL滤后尿液样品用去离子水稀释至10.0 mL，并将尿液样品的pH调至3.0待用。采用PT-SPE技术进行样品净化与富集，在PT-SPE中填装6.0 mg的HMIR-GA吸附剂，依次用1.0 mL甲醇和1.0 mL去离子水活化。随后，加载1.0 mL pH为3.0的尿液样品。用0.8 mL去离子水淋洗，去除水溶性杂质。目标物采用1.4 mL甲醇-乙酸（9∶1，体积比）溶液进行洗脱，收集洗脱液并用氮气吹干，采用0.2 mL的甲醇-0.1%甲酸水溶液（4∶6，体积比）使其充分复溶后采用HPLC进行测定。

### 1.5 色谱条件

赛默飞Acclaim^TM ^120 C18色谱柱（250 mm×4.6 mm，5 μm），流动相为甲醇-0.1%甲酸水溶液（40∶60，体积比），柱温25 ℃，等度洗脱，检测波长278 nm，流速1.0 mL/min，进样体积20 μL。

## 2 结果与讨论

### 2.1 HMIR-GA的制备与优化

HMIR-GA通过表面分子印迹技术在水相环境中合成，合成过程条件温和、绿色环保。氧化石墨烯表面富含大量含氧官能团，既有利于表面接枝反应的进行，又增强了材料在水相样品中的适应性。反应中，绿色环保的交联剂乌洛托品在水解过程中生成的甲醛首先与亲水单体间苯二酚反应生成羟甲基化单体，随后加入虚拟模板*N-*（1-萘基）乙二胺二盐酸盐，与羟甲基化单体形成配对组装。随后，在水热反应下，羟甲基化单体发生缩合反应，并通过共价作用接枝到氧化石墨烯表面，最终形成疏松多孔的HMIR-GA。采用虚拟模板*N-*（1-萘基）乙二胺二盐酸盐可以避免模板渗漏影响肿瘤标志物准确定量的问题。

为获得最佳印迹效果，系统优化了以下反应条件：间苯二酚与乌洛托品的物质的量之比、*N-*（1-萘基）乙二胺二盐酸盐用量、印迹时间以及交联剂聚乙烯醇的用量。间苯二酚与乌洛托品的比例会显著影响所形成亲水性印迹树脂的交联程度，进而影响材料的吸附性能。如图1a所示，当两者的物质的量之比为6∶3时，HMIR-GA对5-HIAA的吸附容量达到最大，因此确定该比例为最优值。

**图1:**
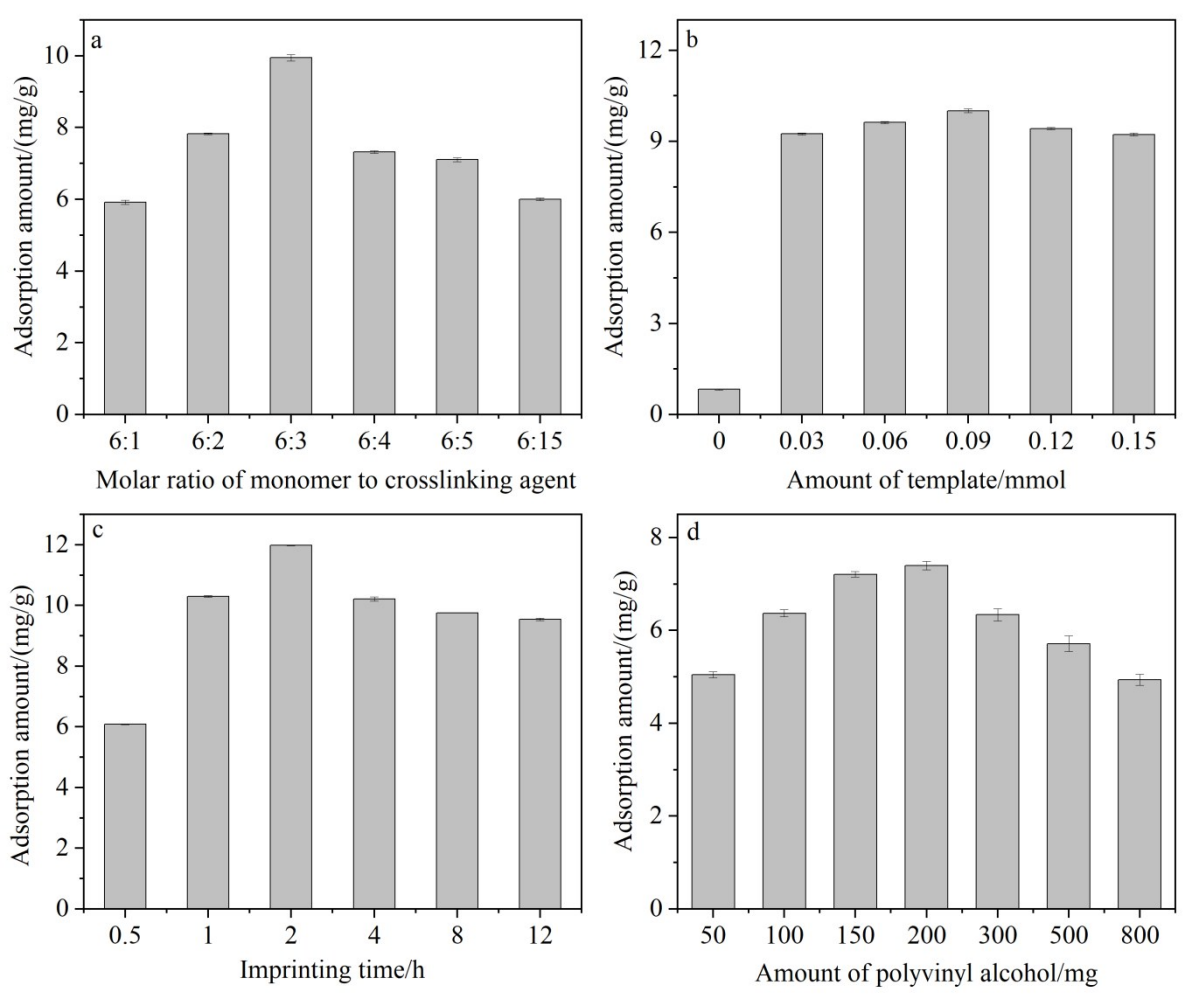
HMIR-GA印迹优化考察图（*n*=3）

*N-*（1-萘基）乙二胺二盐酸盐具有与5-HIAA相似的分子结构，作为虚拟模板有效规避了模板分子泄漏对目标分子定量检测的干扰。在本研究中，系统考察了模板用量对印迹性能的影响。如图1b所示，未添加模板的对照材料HNIR-GA对5-HIAA的吸附量较低。随着虚拟模板用量增加，5-HIAA吸附量逐渐上升，表明印迹位点数量随之增加；当模板用量超过0.09 mmol时，吸附量反而下降。其可能原因包括：模板分子过量会与功能单体竞争，妨碍有序的自组装过程，同时多余的模板难以完全洗脱，占据印迹空穴，从而降低材料的吸附性能^［23，24］^。综上所述，模板的最优用量为0.09 mmol。

印迹时间是决定HMIR-GA对肿瘤标志物特异识别能力的关键因素之一。由图1c可知，随着印迹时间的延长，HMIR-GA对5-HIAA的吸附容量先呈现上升的趋势，这可能是由于当印迹时间小于2 h时，HMIR在GA表面生成的HMIR层较薄，同时HMIR的交联度较低，限制了HMIR-GA的吸附和特异识别能力。然而，当印迹时间大于2 h，HMIR-GA对5-HIAA的吸附量开始下降，这是因为过长的印迹时间会使印迹位点更易包埋于HMIR-GA内部，进而阻碍了其对5-HIAA的吸附和特异性识别性能。因此，本工作选择2 h为最优的印迹时间。

聚乙烯醇含有大量的羟基，其能够与多种目标物形成氢键。因此聚乙烯醇在作为交联剂时具有独特的优势。在石墨烯气凝胶的制备过程中，聚乙烯醇的羟基与石墨烯表面的官能团能够相互作用，从而促进石墨烯片层间的交联与稳定。通过调节聚乙烯醇的用量可以实现对石墨烯气凝胶孔隙结构的精细调控。由图1d可知，随着聚乙烯醇用量的增加，HMIR-GA对5-HIAA的吸附容量增加，当其用量超过200 mg时，其吸附量开始下降，其原因是过量的聚乙烯醇会导致石墨烯气凝胶交联度增强，过度的交联会使印迹位点包埋于HMIR-GA内部，从而影响HMIR-GA对5-HIAA的特异性识别效果。因此，本工作选择最优的聚乙烯醇用量为200 mg。

### 2.2 HMIR-GA的表征

采用扫描电子显微镜（SEM）对制备的HMIR-GA进行形貌与结构表征，从图2a~c可以看出，氧化石墨烯表面具有皱褶的片层结构，而制备得到的HMIR-GA和HNIR-GA呈现疏松多孔结构且褶皱消失，表明制备的HMIR成功地生长在石墨烯气凝胶上，其多孔结构为HMIR-GA提供了更多的吸附位点，从而提高了HMIR-GA对5-HIAA的吸附能力，有利于实现5-HIAA的快速吸附。

**图2:**
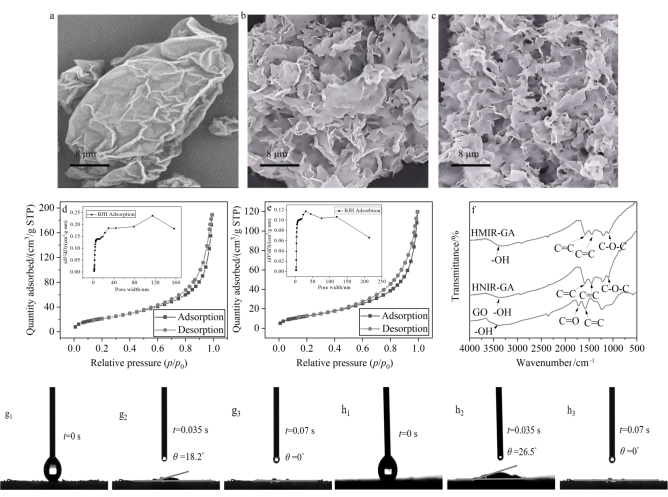
HMIR-GA、HNIR-GA和GO的表征图

采用氮气吸附-脱附实验对材料的比表面积、孔体积和孔径分布进行分析。如图2d、e所示，HMIR-GA与HNIR-GA均呈现多级孔结构，通过BET（Brunauer*-*Emmett*-*Teller）法计算得出，HMIR-GA和HNIR-GA的比表面积分别为95.1 m²/g和44.5 m²/g；孔体积分别为0.31 cm³/g和0.20 cm³/g。进一步利用BJH（Barrett*-*Joyner*-*Halenda）法对孔径分布进行计算，结果显示两者的平均孔径分别为13.7 nm和13.9 nm。HMIR-GA具有较大的比表面积和孔体积，其有利于提供更多的特异性吸附位点，增强了其对肿瘤标志物的识别与吸附能力。

通过傅里叶变换红外光谱（FT-IR）进一步对材料的化学结构进行分析（图2f）。3 400 cm⁻¹处出现的宽峰归属于表面-OH的振动峰，1 724 cm^-1^的吸收峰为氧化石墨烯（GO）表面C=O的伸缩振动峰，由于反应过程中氨水的还原，HMIR-GA和HNIR-GA上的C=O被还原，1 602 cm^-1^和1 462 cm^-1^处的吸收峰为间苯二酚的C=C伸缩振动峰，1 069 cm^-1^处的特征峰是间苯二酚与乌洛托品发生反应生成的C*-*O*-*C基团的伸缩振动峰。FT-IR结果证明HMIR-GA材料成功制备。接触角实验结果（图2g_1_~h_3_）表明，当水滴被分别放置在HMIR-GA和HNIR-GA的表面上时，它在0.07 s内迅速扩散并完全润湿HMIR-GA和HNIR-GA，表明材料具有超亲水性质，有利于石墨烯气凝胶的水相识别能力，提高了对生物样品中肿瘤标志物的吸附能力。

### 2.3 HMIR-GA的吸附性能评价

通过吸附动力学和静态吸附等实验对HMIR-GA的吸附性能进行了系统性评价。从图3a可以看出，在25 ℃条件下，HMIR-GA在240 min内达到吸附平衡，表明其具有较快的吸附速率。从图3b可以看出，随着5-HIAA溶液初始浓度的增加，HMIR-GA的吸附量显著增加。此外，HMIR-GA对5-HIAA的吸附能力随着温度的升高而增加，在318 K时的吸附量高达39.51 mg/g，且HMIR-GA对5-HIAA的吸附量显著高于非印迹材料HNIR-GA，表明其具有更强的亲和性和更优的识别能力。吸附实验结果验证了HMIR-GA在水相体系中对肿瘤标志物的高效吸附与选择识别能力。进一步，本工作对不同批次的HMIR-GA进行了批次间重现性考察，结果表明制备的HMIR-GA批次间重现性良好（RSD=3.9%）。

**图3:**
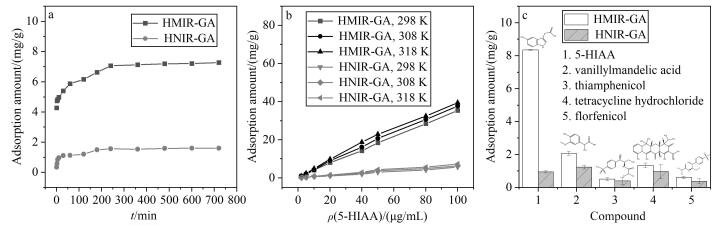
（a）吸附动力学曲线、（b）静态吸附曲线和（c）竞争性吸附结果（*n*=3）

竞争性吸附实验结果见图3c，与其他物质（香草扁桃酸、甲砜霉素、盐酸四环素和氟苯尼考）相比，HMIR-GA对5-HIAA的吸附量显著更高，说明其对目标分子具有优异的特异性识别性能。由表1可知，HMIR-GA对5-HIAA的IF为8.8，进一步表明材料中形成的印迹识别位点具有良好的水相识别能力。综上所述，HMIR-GA展现出优异的吸附容量和选择性识别能力，适用于5-HIAA在水相体系中的高效富集与选择性识别。

**表1:** HMIR-GA的吸附选择性参数

Analyte	*Q*_e_（HMIR-GA）/（mg/g）	*Q*_e_（HNIR-GA）/（mg/g）	Imprinted factor （IF）
5-HIAA	8.34	0.95	8.8
Vanilmandelic acid	2.08	1.24	1.7
Thiamphenicol	0.51	0.42	1.2
Tetracycline hydrochloride	1.34	0.97	1.4
Florfenicol	0.62	0.38	1.6

*Q*_e_： adsorption amount.

### 2.4 HMIR-GA的吸附机理研究

为深入考察HMIR-GA的吸附机理，本研究首先对其吸附动力学行为进行了系统的研究。吸附动力学分析能够帮助预测吸附速率及达到吸附平衡所需的时间。在本工作中，采用准一级动力学模型和准二级动力学模型对数据进行了拟合，以揭示其吸附行为特征。结果表明，HMIR-GA和HNIR-GA对5-HIAA的吸附过程均更符合准二级动力学模型（HMIR-GA和HNIR-GA的*R²*分别为0.999 3和0.998 0），且其拟合吸附量与实际测定值较为接近。这表明该吸附过程主要受化学吸附控制，化学吸附为影响吸附速率的主导步骤^［25］^。

进一步采用Langmuir、Freundlich、Temkin及Dubinin*-*Radushkevich（D*-*R）4种吸附等温模型对5-HIAA在HMIR-GA和HNIR-GA上的吸附过程进行拟合分析。结果显示，两种材料对5-HIAA的吸附均较好地符合Freundlich等温模型（*R²*≥0.985 2），说明其吸附行为更符合非均相表面的多分子层吸附机制。结合吸附等温线分析，可推测5-HIAA在材料表面的吸附过程综合了物理吸附和化学吸附的作用^［26］^。

### 2.5 HMIR-GA-PT-SPE过程优化

首先评估了HMIR-GA用量对尿液样本中5-HIAA损失率的影响规律。从图4a可以看出，随着HMIR-GA用量的增大，5-HIAA的损失率逐渐降低；当HMIR-GA用量达到6.0 mg时，5-HIAA不再损失。为兼顾损失率和处理效率，确定最优的HMIR-GA用量为6.0 mg。

**图4:**
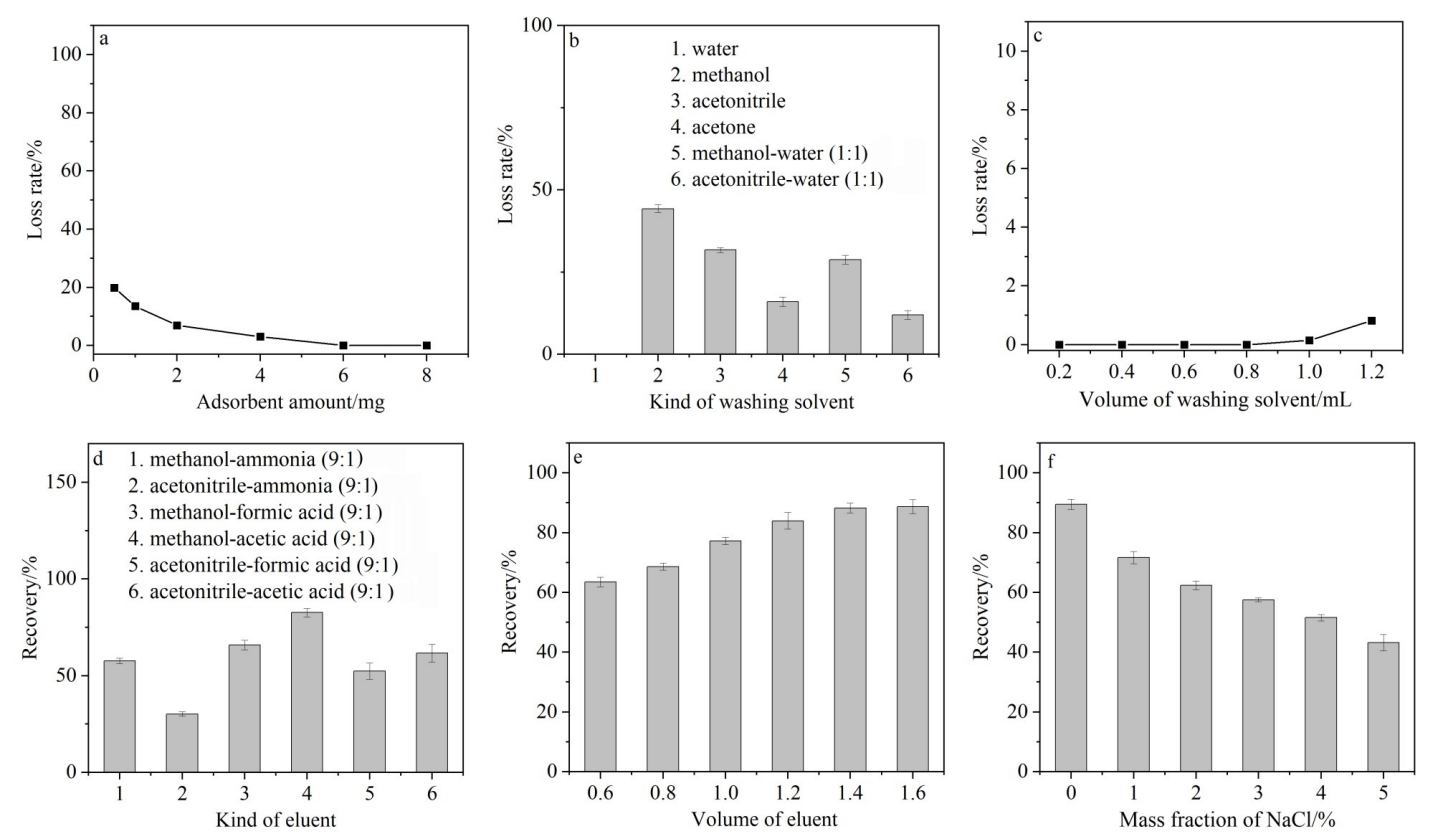
HMIR-GA-PT-SPE过程优化（*n*=3）

为去除吸附在HMIR-GA表面的共吸附杂质，选取6种不同类型的溶剂进行淋洗效果比较。当上样1.0 mL加标尿液样品后，采用1.0 mL各类溶剂进行淋洗除杂。图4b结果表明，当以水作为淋洗剂时，5-HIAA具有最低的损失率，说明水对杂质去除效果优异且不影响目标物的保留。因此选定水作为最优淋洗剂。进一步优化水的用量，如图4c所示，当水的体积为0.8 mL时，5-HIAA的损失率最低，同时尿液样品的净化效果较为理想。因此，本工作最终确定0.8 mL水为最优的淋洗剂用量。

为有效将吸附在HMIR-GA上的5-HIAA解吸附，考察了6种洗脱溶剂的效果。如图4d所示，甲醇-乙酸（9∶1，体积比）展现出最佳洗脱效果。其机制可能原因：乙酸的加入使5-HIAA质子化，从而削弱其与印迹孔穴间的特异性作用力，促进其从吸附剂上脱附。对甲醇-乙酸（9∶1，体积比）的用量进行了优化。如图4e所示，随着洗脱剂体积增加，5-HIAA回收率逐渐上升，在1.4 mL时达到最大，进一步增加体积则回收率趋于稳定。因此，1.4 mL的甲醇-乙酸（9∶1，体积比）为本工作的最佳洗脱剂体积。

尿液样品中的盐浓度可能会对HMIR-GA的萃取效果造成影响，本工作对不同离子强度下HMIR-GA对5-HIAA的萃取效果进行了研究。如图4f所示，随着盐浓度的升高，5-HIAA的回收率呈现逐渐降低的趋势，其原因可能是由于在萃取过程中离子交换作用占据主导地位，而较强的离子作用会抑制尿液中5-HIAA的萃取分离。因此，在后续的HMIR-GA-PT-SPE过程中无需调节其离子强度。

### 2.6 HMIR-GA-PT-SPE-HPLC方法学评价

在最优的HMIR-GA-PT-SPE条件下，对建立的HMIR-GA-PT-SPE-HPLC方法进行了系统评价。首先，向空白尿液样品中分别加入0.02~40.0 μg/mL范围内的5-HIAA标准溶液，制备加标尿液样品，并采用所构建的分析方法进行萃取检测，每个浓度样品平行检测3次。结果表明，在0.02~40.0 μg/mL范围内5-HIAA呈良好的线性关系（*r*=0.999 8），线性方程为*y=*3.156*x+*0.016（*y*为峰面积，*x*为5-HIAA的质量浓度）。根据信噪比分别为3和10的标准，计算得到该方法的检出限和定量限分别为3.7 ng/mL和12.3 ng/mL。为进一步验证方法准确性，选取3个加标水平（0.1、1.0和10.0 μg/mL）进行回收率试验。结果显示，5-HIAA的加标回收率为75.7%~92.5%，RSD均≤3.4%，表明方法具有良好的准确性和重复性。此外，对方法的精密度进行日内和日间考察。结果表明，日内精密度为2.9%，日间精密度为4.1%，进一步验证了所建立方法在不同时间尺度下具有稳定性与可靠性。将所建立的HMIR-GA-PT-SPE-HPLC方法与目前已经报道的方法在线性范围、灵敏度、准确度和精密度方面进行了比较，本研究所开发的方法具有更低的检出限、更高的精密度和更宽的线性范围（表2）。综上，本工作所建立的HMIR-GA-PT-SPE-HPLC方法具有良好的灵敏度、准确度与精密度，适用于复杂尿液样品中肿瘤标志物5-HIAA的高效分离与富集检测。

**表2:** 与文献中已有检测方法的比较

Method	Linear range/（μg/mL）	LOD/（ng/mL）	Recoveries/%	RSD/%	Ref.
LC-MS/MS	0.1-8.3	32.3	87.1-107.0	≤6.6	［^5^］
GC-QqQ-MS	0.5-100	24.3	91.3-106.6	≤8.9	［^6^］
HPLC-ECD	0.5-19.1	765	85.8-90.7	≤7.1	［^27^］
HPLC-ECD	3.8-28.7	57.4	92.0-95.0	≤17.4	［^28^］
HPLC-UV	0.02-40	3.7	75.7-92.5	≤3.4	this work

QqQ： triple quadrupole； ECD： electrochemical detection.

### 2.7 HMIR-GA与商业化材料对比

采用5-HIAA的回收率为评价指标，将制备的HMIR-GA和HNIR-GA与C_18_、HLB、WAX、MCX等商业化吸附剂进行对比。HMIR-GA和HNIR-GA采用优化后的最佳萃取条件进行萃取，商业化吸附剂采用文献中报道的方法进行萃取过程^［29-32］^。结果如图5a所示，采用HMIR-GA作为吸附剂时，对尿液中5-HIAA具有最高的回收率，其原因是HMIR-GA与5-HIAA之间具有氢键、*π*-*π*共轭等作用，亲和力更强，回收率更高。此外，HMIR-GA具有独特的印迹孔穴，对复杂尿液样品具有明显的净化效果。

**图5:**
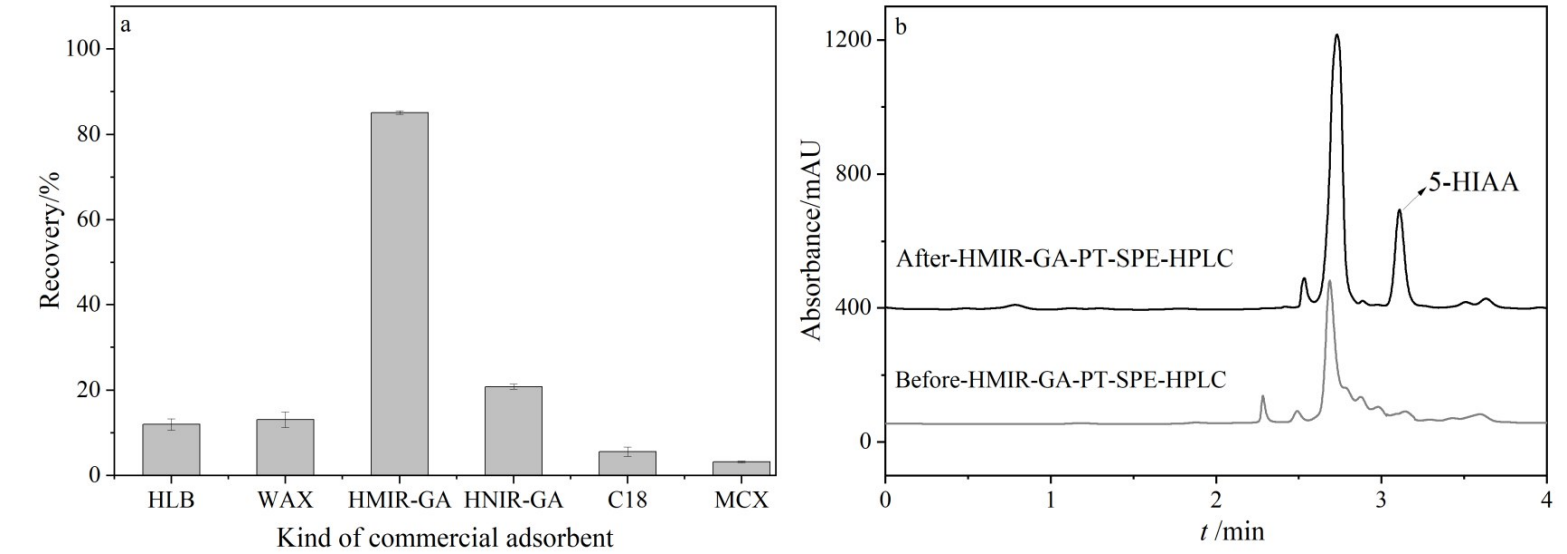
（a）与商业化材料对比图（*n*=3）和（b）尿液样品的色谱图

### 2.8 HMIR-GA-PT-SPE-HPLC方法实际应用

将建立的HMIR-GA-PT-SPE-HPLC方法应用于实际尿样中肿瘤标志物5-HIAA的检测。由图5b可见，经HMIR-GA吸附净化后，5-HIAA的色谱峰保留时间区域未出现其他干扰峰，说明尿液基质组分对5-HIAA的检测基本无干扰，具有优异的净化能力，验证了该方法对5-HIAA的良好选择性和准确定量能力。为进一步评估该方法在实际样品中的适用性，对6位志愿者的尿液样品进行了检测，其含量为3.07~5.91 μg/mL，表明该方法可以实现尿液样品中痕量肿瘤标志物的快速精准检测，具有较好的实际应用潜力。

## 3 结论

本研究成功制备了一种新型超亲水印迹复合材料HMIR-GA，其具有优异的水相识别能力和较高的吸附容量。在此基础上建立了基于HMIR-GA的管尖固相萃取-高效液相色谱法，用于生物样品中肿瘤标志物的选择性萃取富集与定量分析。吸附实验表明，HMIR-GA具有较高的吸附容量和良好的分子识别选择性，能够有效区分目标分子与共存干扰物。所建立的HMIR-GA-PT-SPE-HPLC方法具有灵敏度高、重现性好等优点，成功应用于生物样品中肿瘤标志物的精准检测，验证了其实际应用价值。该新型亲水分子印迹材料为复杂生物样品中肿瘤标志物的选择性萃取和检测提供了新的技术思路，具有良好的应用前景。
